# Emerging rejuvenation strategies—Reducing the biological age

**DOI:** 10.1111/acel.13538

**Published:** 2021-12-31

**Authors:** Bohan Zhang, Alexandre Trapp, Csaba Kerepesi, Vadim N. Gladyshev

**Affiliations:** ^1^ Division of Genetics Department of Medicine Harvard Medical School Brigham and Women’s Hospital Boston Massachusetts USA

**Keywords:** aging, biomarkers, epigenetic clocks, rejuvenation

## Abstract

Several interventions have recently emerged that were proposed to reverse rather than just attenuate aging, but the criteria for what it takes to achieve rejuvenation remain controversial. Distinguishing potential rejuvenation therapies from other longevity interventions, such as those that slow down aging, is challenging, and these anti‐aging strategies are often referred to interchangeably. We suggest that the prerequisite for a rejuvenation intervention is a robust, sustained, and systemic reduction in biological age, which can be assessed by biomarkers of aging, such as epigenetic clocks. We discuss known and putative rejuvenation interventions and comparatively analyze them to explore underlying mechanisms.

## Graphical abstract

Emerging interventions have suggested that biological aging can be reversed rather than just attenuated, but the criteria for what it takes to achieve rejuvenation remain controversial. In this review, we discuss known and putative rejuvenation interventions, exploring their connections within the context of aging biomarkers. We suggest that the prerequisite for classifying an intervention as rejuvenating is a robust and reproducible reduction in biological age, which can be assessed by high‐dimensional trackers of aging such as epigenetic clocks.

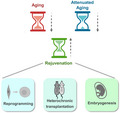

**Abbreviations**
AAV: adeno‐associated virus
CRISPR: clustered regularly interspaced short palindromic repeats
iPSCs: induced pluripotent stem cells iPSC

## 1 INTRODUCTION

As the most significant risk factor for human mortality, aging leads to functional decline, increased frailty, and elevated susceptibility to chronic disease (Brett & Rando, 2014; Lopez‐Otin et al., 2013). The current strategies for human lifespan extension can be divided into three major categories: (i) those that treat direct causes of mortality, (ii) those that slow down or attenuate the biological aging process, and (iii) those that achieve rejuvenation (i.e., the reversal of aging). The first category involves treatments for age‐related diseases, such as pharmaceuticals for COVID‐19 in humans or age‐related cancers in mice. Antibiotics, which single‐handedly shifted the main cause of death in humans and extended lifespan by several decades, also belong to this category (Adedeji, 2016). The second involves lifespan extension in healthy individuals, without evident age reversal. One example in this category is lifespan extension caused by mild stressors such as heat, cold, or irradiation (Cypser et al., 2006; Gems & Partridge, 2008). The third category, rejuvenation, has long been regarded as the panacea for age‐related diseases, but it has previously been deemed unrealistic. While the first two major strategies have been extensively studied, very little is known about the systemic reversal of organismal aging. This is in part due to the lack of longitudinal data and validated quantitative readouts of rejuvenation, and also by the general belief that aging is inevitable and unidirectional. However, several putative rejuvenation therapies have recently been introduced that demonstrated age reversal as measured by aging biomarkers and physiological readouts. Despite these advances, whether systemic rejuvenation can be achieved by these approaches and how they can be translated to human applications remains unclear. To distinguish rejuvenation therapies from other longevity interventions, it is necessary to establish a framework that describes different approaches to rejuvenation while exploring the critical common underpinnings of established age reversal methods.

### 1.1 Challenging the notion of irreversible aging

Aging of mammalian species, such as humans or mice, has traditionally been regarded as an irreversible process. This is largely due to the commonly held belief that certain tissues, cells, and structures in these organisms are irreplaceable (Galkin et al., 2019). For example, most adult neurons terminally differentiate during development, remain in the body for the entire life of an organism, and cannot be naturally replaced. Interestingly, in some non‐mammalian species or in young mammals, certain body parts or organs can be regenerated or regrown in almost identical structure as the lost tissue (e.g., axolotl extremities (Haas & Whited, 2017) and newborn murine heart tissue (Bryant et al., 2015)). However, adult humans lack sustained cross‐tissue regenerative capacity.

This notion of irreversibility has recently been challenged by a series of findings. With the in vivo ectopic expression of Oct4, Sox2, and Klf4—three of the four Yamanaka reprogramming factors (Takahashi & Yamanaka, 2006)—axon regeneration after eye injury has been achieved. Excitingly, this strategy also allows mice to regain eyesight lost as a result of aging or glaucoma (Lu et al., 2020). In addition, a drug cocktail has shown potential for thymus regeneration, further challenging the idea of unidirectional aging (Fahy et al., 2019). However, the overwhelming majority of these potential rejuvenation therapies have so far focused on specific organs or sets of tissues; therefore, the effect of rejuvenation on systemic aging has not yet been well described. More importantly, the lack of robust biological age quantification methods—until the recent emergence of molecular aging biomarkers—has confined characterization of rejuvenation mostly to visual or functional investigation of tissue‐specific aging phenotypes.

To clearly define the effects of rejuvenation and characterize them systemically, there needs to be both an initial, accurate assessment of biological age coupled with subsequent quantification of biological age dynamics in response to these putative interventions. Here, we define organismal rejuvenation as a robust, sustained, and systemic decrease in biological age or damage, measured by accurate physiological and/or molecular biomarkers. Under this definition, the majority of validated and putative rejuvenation interventions and phenomena may be categorized into three main groups: (i) heterochronic transplantation, (ii) cellular reprogramming, and (iii) early embryonic dynamics. In this review, we elaborate on this definition of rejuvenation, its three current categories, and the principal differences and commonalities among them.

### 1.2 Biomarkers that track the reversal of aging

First, it is important to note that the reversal of aging is inherently multidimensional: it may include a reduction in damage at the molecular level, renewed cell functionality at the cellular level, and meaningful physiological improvement at the organismal level. Some age reversal therapies may also induce lifespan extension, unless limited by extrinsic mortality factors, such as high tumor incidence in mice at old ages (Brayton et al., 2012; Turturro et al., 2002). Fundamentally, the effect at one level of biological organization is usually accompanied by connected effects at other levels. As an example, expression of reprogramming factors OSK (Oct4 + Sox2 + Klf4) or OSKM (OSK + c‐Myc) was shown to reverse epigenetic age, increase stem cell function, reverse age‐related loss of eyesight, and increase lifespan of progeria mouse models. In fact, interventions usually influence age‐related phenotypes across multiple levels, and robust measures (biomarkers) of age‐related damage at one level could be used to identify putative rejuvenation interventions.

One of the critical issues in distinguishing rejuvenation from other longevity interventions remains longitudinal examination of aging biomarkers to reveal a steady decrease or reversal in biological age throughout the whole intervention procedure, and beyond. This inherently requires biomarkers to be noninvasive or at least nonlethal, and many studies at the tissue level are restricted by this criterion (organs can only be harvested from sacrificed mice at one given timepoint). However, at the molecular and physiological level, there are several robust biomarker profiling methods available. From a physiological standpoint, the frailty index has been established as a powerful tool to assess biological age, and the clock based on it has been successfully used to evaluate methionine restriction as a longevity intervention. (Schultz et al., 2020; Whitehead et al., 2014). However, this method is intrinsically subjective and is meant to be applied primarily to aged animals, limiting its use for assessment of biological age reversal in young animals.

At the molecular level, several omics‐based approaches for quantifying aging and rejuvenation have emerged. Indeed, it is now possible to assess biological age using a variety of high‐dimensional molecular data, particularly through the implementation of sophisticated shallow and deep machine learning methods into what is known as “biological aging clocks”. So far, several methods have been developed to examine age‐related molecular dynamics, including clocks focusing on the epigenome, transcriptome, and immunome (Galkin et al., 2020, 2021; Horvath, 2013; Meyer & Schumacher, 2021; Sayed et al., 2021). Among these methods, aging biomarker models based on DNA methylation (termed “epigenetic aging clocks”) have emerged as some of the most promising methods, with diverse applications across mammalian species (Bell et al., 2019; Hannum et al., 2013; Horvath & Raj, 2018; Petkovich et al., 2017). Multiple clocks have been developed for humans, including multi‐tissue (Horvath, 2013) and single‐tissue clocks (Hannum et al., 2013), as well as the PhenoAge and GrimAge clocks that predict health span, lifespan, and mortality risks (Levine et al., 2018; Lu et al., 2019). Moreover, epigenetic aging clocks have been developed across various tissues, platforms (most commonly DNA microarrays or genome‐wide/targeted bisulfite sequencing approaches), type of DNA sequences (genomic or ribosomal DNA), and model species (such as humans, mice, and rats) (Horvath et al., 2020; Li et al., 2021; Lu et al., 2021; Meer et al., 2018; Petkovich et al., 2017; Stubbs et al., 2017; Trapp et al., 2021; Wang & Lemos, 2019; Wang et al., 2017). Excitingly, novel clocks have also been developed that track aging across most eutherian species with a single mathematical formula, demonstrating the universal and generalizable nature of cross‐species epigenetic alterations with age (Lu et al., 2021).

Crucially, epigenetic age predictions from methylation clocks in model species reflect various lifespan‐extending treatments such as caloric restriction and growth hormone receptor knockout (Petkovich et al., 2017). Additionally, epigenetic clocks were shown to quantitatively measure several aspects of human aging: indeed, epigenetic age acceleration was associated with many age‐related conditions, such as all‐cause mortality, cognitive performance, frailty, progeria, Parkinson's disease, Werner syndrome, and Hutchinson Gilford Progeria Syndrome (Breitling et al., 2016; Horvath et al., 2018; Horvath & Raj, 2018; Lin et al., 2016; Maierhofer et al., 2017; Marioni, Shah, McRae, Chen, et al., 2015; Marioni, Shah, McRae, Ritchie, et al., 2015). Taken together, these studies offer evidence that aging clocks based on methylation levels may accurately track biological age and act as validators of putative rejuvenation therapies (Horvath, 2013; Petkovich et al., 2017). Excitingly, other transcriptomic, ionomic, compositional, and frailty clocks have recently shown promise for high‐resolution tracking of the aging process and biological age alterations resulting from longevity or rejuvenation interventions (Meyer & Schumacher, 2021; Putin et al., 2016; Zhang et al., 2020).

Despite rapid progress in the field of epigenetic aging clocks, it should be noted that there are still many important unknowns, the most critical of which is the directionality and extent of the relationship between epigenetic changes, molecular damage, and biological age. Indeed, it is believed that DNAm age prediction may be readout of the molecular damage involved in aging. However, it remains enigmatic whether epigenetic changes are themselves damage, or simply a reflection of other molecular damage. Of note, recent work has shown that double‐stranded breaks may contribute to aging and epigenetic drift (Hayano et al., 2019). Additionally, the reproducibility of DNAm measurement can be restricted by technical noise, which has been recently addressed (Higgins‐Chen et al., 2021).

Importantly, although changes in DNAm age may be used for preliminary identification of novel rejuvenation therapies, these must still be evidently validated across several other biological levels, and particularly at the phenotypic/physiological level. For example, simply editing CpG methylation levels of certain clock sites to “young” levels is unlikely to change physiological functions, but this is still unknown. New methods enabling targeted epigenomic remodeling via CRISPR‐based approaches may hold the key to definitely answer this complex question (Nunez et al., 2021).

**FIGURE 1:**
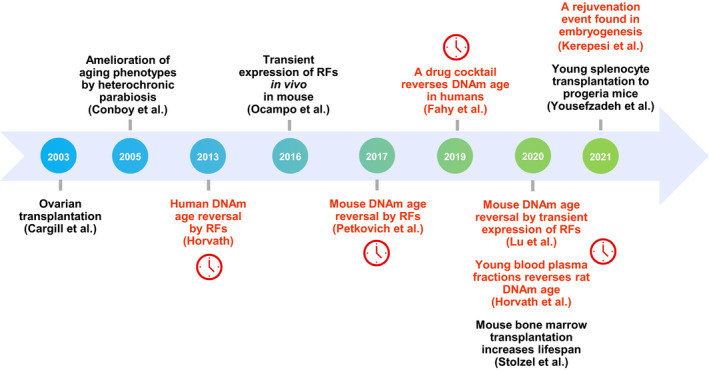
Timeline of advances in rejuvenation research. Several potential rejuvenation therapies that fall into the three major categories are listed in chronological order. Treatments marked in red show a reversal in biological age as assessed by epigenetic clocks. Additional studies not shown on this timeline are described in Table 1. RF, reprogramming factor

Despite these unanswered questions, DNA methylation clocks have emerged as a powerful set of tools to identify potential rejuvenation therapies. It has become possible to evaluate and test several existing approaches aiming to reverse biological age (Figure 1 and Table 1). In humans, the thymus regeneration strategy mentioned previously has been shown to lower epigenetic age, supporting the possibility that certain drug interventions may rewind biological age (Fahy et al., 2019). However, these results are still preliminary, and it should be noted that this study had a small sample size (*n* = 10) and no control group. Other popular interventions focus on the expression of reprogramming factors, which were shown to directly reverse epigenetic age of adult cells to zero in cell culture systems (Horvath, 2013; Olova et al., 2019; Petkovich et al., 2017). A promising milder expression strategy, known as transient or partial reprogramming, involves reversing biological age of multiple cell types while maintaining cell identity (Gill et al., 2021; Ocampo et al., 2016). When this strategy is applied in vivo, reprogramming factors have not only shown lifespan extension of animals with a progeroid syndrome, but also demonstrated the ability to rejuvenate retinal cells through neuro‐regeneration (Lu et al., 2020; Ocampo et al., 2016). Additionally, treatment of plasma fraction combinations was shown to reverse epigenetic age in rats (Horvath et al., 2020). Together, these studies point to the notion that aging may be reversible via a variety of approaches.

**TABLE 1:** Studies reporting biological age reduction

Study	Rejuvenation class	Species	Accession	Clock(s) applied	Reported biological age reduction
Horvath (2013)	Reprogramming (in vitro)	Human	GSE30653 GSE31848 GSE38806	Horvath multi‐tissue	iPSCs have a lower DNAm age than corresponding primary cells
Petkovich et al. (2017)	Reprogramming (in vitro)	Mouse	GSE80672	Petkovich blood	iPSCs have a lower DNAm age than corresponding primary fibroblasts
Olova et al. (2019)	Reprogramming (in vitro)	Human	GSE54848	Horvath multi‐tissue Weidner 99 CpG Skin & blood PhenoAge Hannum blood Weidner 3 CpG	Steady decrease in epigenetic age during reprogramming of fibroblasts reported by 3 of the 5 applied epigenetic clocks (two other clocks did not show informative trajectories)
Meer et al. (2018)	Reprogramming (in vitro)	Mouse	GSE80672	Meer multi‐tissue Stubbs multi‐tissue Petkovich blood Wang liver	iPSCs have remarkably lower DNAm age than primary fibroblasts as shown by 2 of 4 epigenetic clocks (minimal change by two other clocks)
Wang and Lemos (2019)	Reprogramming (in vitro)	Mouse	GSE80672	Wang blood rDNA	iPSCs have a lower ribosomal DNAm age than primary fibroblasts
Fahy et al. (2019)	Thymus regeneration treatment (in vivo)	Human	NA	Horvath multi‐tissue PhenoAge Hannum blood GrimAge	A decrease in epigenetic age after 12 months of treatment (intended to regenerate the thymus) by four applied clocks
Sarkar et al. (2020)	Reprogramming (in vitro)	Human	GSE142439	Horvath multi‐tissue	Transient reprogramming reverted the DNA methylation age of aged fibroblasts and endothelial cells
Lu et al. (2020)	Reprogramming (in vivo)	Mouse	PRJNA655981	Wang blood rDNA	Lower rDNAm age of RGCs from axon‐injured retinas upon an OSK treatment
Horvath et al. (2020) (preprint)	Heterochronic transplantation (in vivo)	Rat	NA	5 rat clocks (pan‐tissue, blood, liver, heart, brain) Human‐rat	Lower epigenetic age after a plasma fraction treatment in four tissues
Gill et al. (2021) (preprint)	Reprogramming (in vitro)	Human	NA	Horvath multi‐tissue Skin & blood Transcriptome	Remarkable (~30 year) decrease in epigenetic age and transcriptomic age by maturation phase transient reprogramming of fibroblasts
Kerepesi et al. (2021)	Reprogramming (in vitro)	Mouse	GSE80672	Multi‐tissue rDNA	iPSCs have a lower DNAm age than primary fibroblasts
	Embryonic (in vivo)	Mouse	GSE34864 GSE56697 GSE98151 GSE121690 GSE51239	Petkovich blood Stubbs multi‐tissue Meer multi‐tissue Thompson multi‐tissue Blood rDNA Multi‐tissue rDNA	Epigenetic age of embryonic day 6.5/7.5 embryos is lower than in earlier stages of embryogenesis by all of applied clocks
Trapp et al. (2021) (preprint)	Embryonic (in vivo)	Mouse	GSE121690	scAge	Profound epigenetic age decrease in single cells between embryonic days 4.5 and 7.5

Abbreviations: iPSC, induced pluripotent stem cell; NA, not available; OSK, *Oct4*/*Sox2*/*Klf4*; rDNA, ribosomal DNA; RGC, retinal ganglion cell.

Although several interventions assessed by aging clocks have shown an age reversal effect, many remain to be tested. Of the established and promising interventions, heterochronic transplantation, youthful factor expression, and age reversal during embryogenesis are three particularly interesting facets with putative *in vivo* applicability in human systems. Understanding key links between these approaches may help to elucidate the underlying mechanisms of age reversal, eventually enabling development and application of robust rejuvenation therapies in humans.

## 2 REJUVENATION BY HETEROCHRONIC TRANSPLANTATION

It has long been known that tissues and organs from animals of one age can be transplanted to animals of different ages to form “heterochronic” age chimeras (Krohn, 1962). Of the potential rejuvenation therapies that remain to be thoroughly characterized, heterochronic parabiosis is one of the most notable. This surgical procedure has been performed for years on rodents, and it was shown that mouse lifespan can be extended by linking the circulatory system of an old mouse with that of a young mouse (Ludwig & Elashoff, 1972). Heterochronic parabiosis was rediscovered as one of the most promising rejuvenation interventions in 2005 (Conboy et al., 2005). By briefly connecting the circulatory system of young and aged mice, old mice exhibited youthful features in the brain, muscle, and liver, characterized by increased cognitive function, replenished stem cell pools, and augmented regenerative capacity.

**FIGURE 2:**
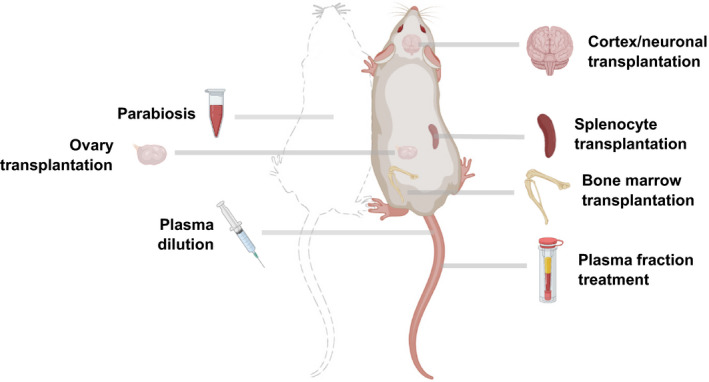
Heterochronic transplantation in mouse models. Schematic of potential heterochronic transplantation interventions for rejuvenation in mice

Following up on this study, researchers have focused on blood components for biological age reversal, while others have investigated transplantation of other organs types to replace aged tissues (Figure 2). With bone marrow transplantation, the blood epigenetic age of the recipients generally matches the age of the donors, although whether this effect is systemic has yet to be established (Stolzel et al., 2017). Of note, bone marrow transplantation has also shown a 12% increase in mouse lifespan (Guderyon et al., 2020). Additionally, a study using an undisclosed plasma fraction demonstrated robust reversal of epigenetic age, further suggesting that heterochronic transplantation may be a potential rejuvenation intervention (Horvath et al., 2020). Recent work has also revealed that young splenocyte transplantation ameliorates aging features of progeroid animals (Yousefzadeh et al., 2021). In addition, transplantation of embryonic brain tissues has shown potential for neuronal repair (Falkner et al., 2016; Gaillard et al., 2007; Hebert & Vijg, 2018), and ovarian transplantation was reported to improve health parameters and result in elongated lifespan (Cargill et al., 2003; Mason et al., 2009).

Following this set of promising experiments, a key question about their underlying mechanisms emerges: are the effects observed caused by the dilution or removal of age‐related detrimental factors, or do they alternatively occur as a result of introducing beneficial youthful factors (i.e., juvenile protecting factors)? Research on youthful factor introduction as an age reversal therapy has focused on identifying the active component(s) that serve as rejuvenation factors in parabiosis, resulting in part in the discovery of the effect of oxytocin on regenerative capacity (Elabd et al., 2014). Consistent with these results from heterochronic bone marrow transplantation and plasma treatment experiments, circulation of young blood immune cells and/or specific components of plasma may be a potential mechanism for the reversal of biological age. Importantly, the possibility that individual components have a certain longevity effect is not in conflict with the thought that aging occurs systemically, although the existence of such component is yet to be validated. Instead of directly targeting certain disease‐related genes or proteins, these components will likely act globally to extend lifespan and slow aging independent of targeting disease, and although the endpoint‐elongation of lifespan may be the same, the way in which this longer life is achieved is different from a clear pharmaceutical treatment.

While some discoveries emphasize the importance of young blood components, others highlight the importance of diluting “old factors” or damage to achieve rejuvenation. This approach is consistent with the idea that detrimental bio‐molecules, such as side‐products of intracellular biochemical reactions, accumulate with age in the body (Gladyshev, 2016; Zhang & Gladyshev, 2020). Aging is thought to involve an inevitable increase in a myriad of biochemical damage forms that arise as by‐products of metabolism and inefficiencies in maintenance pathways. This damage is incompletely cleared by processes such as DNA repair and detoxification pathways at the molecular level, autophagy at the subcellular level, and senescence/apoptosis at the cellular level. Within this framework, the accumulation of damage is thought to subsequently cause age‐related phenotypes, manifesting in decreased cell functionality and physiological frailty. Hence, it is possible that lowering the concentration of damage via dilution could partially restore youthful functional features. Consistent with this idea, it has been recently reported that diluting aged plasma with saline and albumin contributes to muscle repair (Mehdipour et al., 2020).

Some may argue that dilution of old factors better categorizes as a longevity intervention than a rejuvenation therapy. A simple thought experiment can be brought into play to consider this idea. Let us imagine a single cell that starts of with no biological damage; hence, a biological age of 0. Now, let us also consider that damage takes only one form, perhaps the accumulation of a specific molecule or by‐product. As the cell proceeds with its life, it will inevitably accumulate several such “damaging” molecules. Now, let us imagine that the cell reaches a point where it is heavily damaged, quantified as having an arbitrarily high number of damaging molecules. Thinking about the accumulation of damage as the principal marker of “age” in this simplistic cell model, if the effective concentration of these damaging molecules was somehow halved in a cell, perhaps through dilution or other mechanisms, it would follow that the cell is now only half as old. Hence, its lifespan has not only been extended, but it has truly undergone a rejuvenation or age reversal event. Of course, this is just simplistic thought experiment, but it may help to understand the putative complex interplay between damaging factors, aging, and rejuvenation.

It is important to note that these two viewpoints—damage removal and youthful factor introduction—are far from mutually exclusive. The global effect observed in several studies may in fact be due to an interactive combinatorial effect of both (1) introducing juvenile factors and (2) diluting/removing damage. In future, it will be critical to separately assess the effects of damage dilution and youthful factor introduction on systemic age reversal in humans and in model organisms.

### 2.1 Rejuvenation by expression of reprogramming factors

On a microscopic scale, the most extreme case of heterochronic transplantation is somatic cell nuclear transfer, which has emerged as the basis for modern‐day cloning approaches (Gurdon et al., 1958). By transferring an adult cell nucleus to a de‐nucleated oocyte, a new individual can be generated. This technique encapsulates the full potential of reversing the biological age of a somatic cell to that of the new embryo (Wilmut et al., 1997). Interestingly, this implies that methylation patterns in the transferred nucleus are likely reset by cytosolic components in the oocyte, suggesting another potential mechanism for rejuvenation.

To recapitulate this effect without the introduction of complex microscopic procedures, scientists discovered four critical “reprogramming factors” (Yamanaka factors), which when expressed in somatic cells, could effectively reverse the developmental status to that of early embryos, generating induced pluripotent stem cells (iPSCs) (Takahashi et al., 2007; Takahashi & Yamanaka, 2006). Interestingly, when epigenetic clocks were applied to iPSC samples, low epigenetic ages around zero were predicted (Horvath, 2013; Petkovich et al., 2017). Several clock models—Horvath multi‐tissue (Horvath, 2013), Weidner 99 CpG (Lin et al., 2016), Skin & Blood (Horvath et al., 2018), PhenoAge (Levine et al., 2018), Hannum blood (Hannum et al., 2013), Weidner 3 CpG clock (Weidner et al., 2014)—reported the application of epigenetic clocks to iPSC reprogramming (Olova et al., 2019). Almost all clocks showed considerable epigenetic age decreases compared with dermal fibroblasts used as the source of fully reprogrammed iPSCs. On the contrary, the clocks exhibited considerable variability in the actual epigenetic age of iPSCs, which ranged from −60 to 10 years. Similar characteristics were observed in mice. Five epigenetic clocks were applied to fibroblasts from adult mice and derived iPSCs (Meer et al., 2018; Petkovich et al., 2017). Three of these clocks showed a remarkable decrease in epigenetic age after reprogramming, while two clocks showed minimal changes. In the case of mice, clocks reported a range of epigenetic ages of the same iPSCs from −1.4 to 3 months. Taken together, most human and mouse clocks reach a general consensus in establishing age reversal that occurs as a result of reprogramming, although consistent predictions of age in these cells across different models remain a challenge.

Ultimately, these observations are in line with the fact that iPSCs can contribute to the whole new embryo, corroborating reprogramming as a key rejuvenation intervention (Kang et al., 2009). With this in mind, several studies have applied these approaches in vivo. By modulating transient expression of reprogramming factors in the whole body to avoid cells being fully reprogrammed to iPSCs, researchers were able to extend the lifespan of progeria mice and improve tissue repair (Ocampo et al., 2016). A similar approach using adeno‐associated virus (AAV) induction achieved the reversal of epigenetic age as well as neuronal regeneration in the retina (Lu et al., 2020). It has also been reported that reprogramming factor expression in human muscle cells can enhance functional restoration of the muscle stem cell reservoir (Sarkar et al., 2020). Together, these findings strongly support the idea that systemic rejuvenation may occur when reprogramming factors are expressed.

One crucial caveat to this approach is whether reprogramming can be carefully modulated to harness age reversal while preventing unwanted effects. Conventional reprogramming methods totally alter cell lineage and identity, and as a result, cells lose their structure and functions. When this full reprogramming method is applied to mice in vivo, it promotes tumor formation, interfering with the rejuvenation effect (Abad et al., 2013; Ohnishi et al., 2014). It has been shown in cell culture studies that human cells may achieve epigenetic age reversal without changing cell lineage markers (Gill et al., 2021). However, as the four reprogramming factors (Oct4, Sox2, Klf4, and c‐Myc) create a black box of mixed downstream gene expression patterns, the sets of genes specifically responsible for biological age reversal have yet to be distinguished from the genes responsible for de‐differentiation and reversal of cell identity. Although partial reprogramming methods have been developed to minimize the tumorigenic effect, the risk has yet to be totally controlled for. Therefore, a potential future effort might be to investigate intrinsic connections and differences between the reversal of cell identity and the reversal of biological age.

**FIGURE 3:**
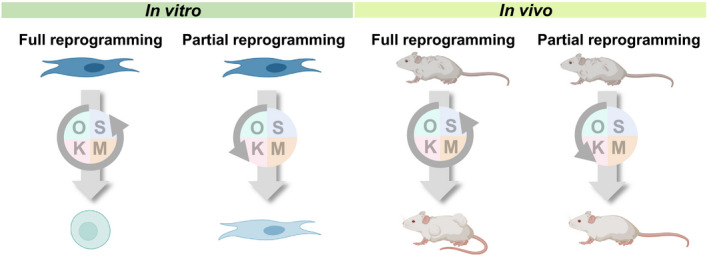
Reprogramming approaches for rejuvenation. Schematic of reprogramming approaches for rejuvenation in vitro and in vivo. Full reprogramming of cells in vitro can reverse biological age to that of the embryo, but this approach can be tumorigenic in vivo. Partial reprogramming could reverse biological age of the cell without an irreversible change of cell identity, and the in vivo approach may be promising in order to achieve rejuvenation

In this regard, a key potential experiment would be to study the fundamental intersection of downstream gene expression dynamics common to OSK/OSKM expression and other interventions or biological events that cause robust reversal of aging assessed by molecular aging biomarkers. This would enable investigation of alternative reprogramming factors downstream of OSKM that only reverses the molecular aging biomarker readout and age‐related cellular phenotypes, but not cell identity (Figure 3). Alternatively, it would be extremely interesting to identify genetic programs that lead only to loss of cell identity, but not biological age reversal. Furthermore, it is worth noting that reprogramming factors were originally identified by screening embryonic‐specific gene expression patterns. Hence, the dynamics of biological age in embryos may provide insights into novel rejuvenation therapies.

### 2.2 A rejuvenation event during early embryonic development

There is a profoundly evident contradiction in the concept of irreversible aging, which is the reset of individual age from generation to generation. This reset precisely sets the clock back to zero, guaranteeing that a species does not die out due to a constant biological age increase as a function of the passage of generations. It was recently proposed that germline cells may age and be rejuvenated in the offspring after conception (Ashapkin et al., 2017; Gladyshev, 2021). If this is the case, there must be a point (or period) of the lowest biological age (here, referred to as “ground zero”) during the initial phases of embryogenesis. Indeed, by applying an array of established and novel DNA methylation clocks to study embryonic development in mice and humans, a natural rejuvenation event was identified during mid‐embryogenesis followed by organismal aging (Kerepesi et al., 2021; Trapp et al., 2021). This rejuvenation event results in the lowest biological age of the organism, occurring approximately at the stage of gastrulation. Additionally, single‐cell analyses revealed that the rejuvenation effect observed in early embryogenesis is stratified to certain cell lineages, wherein supportive extra‐embryonic cell lineages do not appear to undergo rejuvenation. Notably, this rejuvenation event is also reflected in ribosomal DNA methylation dynamics, indicating that ribosomal DNA likely accumulates damage and may be rejuvenated during embryogenesis. This notion is particularly interesting within the context of previous findings that perturbations in ribosomal expression dynamics affect gamete health (Duncan et al., 2017). Also, a renewal of oocyte proteostasis was observed in *Caenorhabditis elegans* before fertilization, induced by sperm cells (Bohnert & Kenyon, 2017). This by itself may be considered another germline‐related rejuvenation process. It is also important to note that gametes are not immune to age‐relate damage‐they do age. Therefore, it would be particularly interesting to find out how gametes are affected by these cellular maintenance machineries (Cao et al., 2020).

**FIGURE 4:**
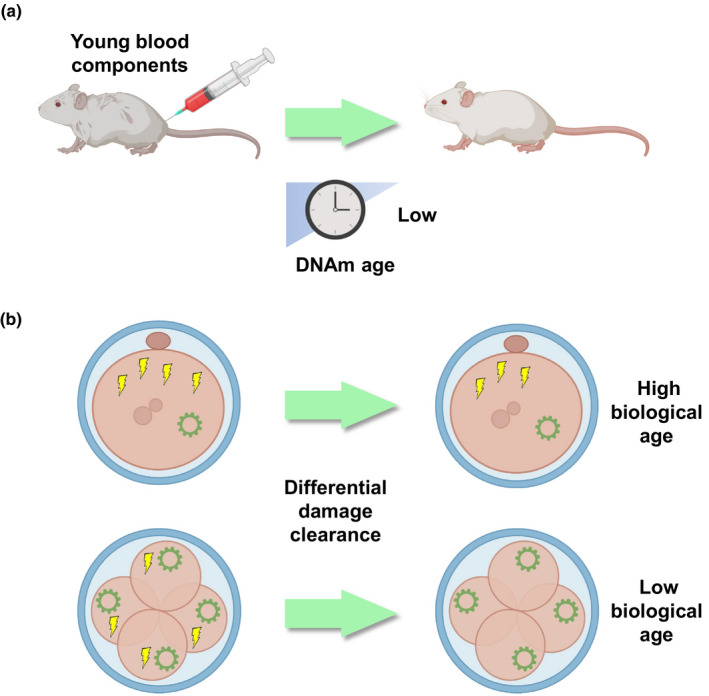
Damage dilution in rejuvenation. Schematic of damage dilution, a potential mechanism shared between embryonic rejuvenation and heterochronic transplantation. In (a), heterochronic transplantation, the damage accumulated with age is likely diluted by donor tissues (i.e., young blood), resulting in lower DNAm age readouts. In early embryonic development (b), this is done through cell division, unequal distribution of damage, and an increase in cellular maintenance and repair machinery. In a single cell, damage can only be cleared at a certain rate, depending on the abundance of maintenance mechanisms (top). During highly proliferative states (bottom), damage is distributed (likely unequally) to different cells, which can each handle the reduced damage with their own repair tools. Bulk and single‐cell clocks may be used to assess biological age readouts resulting from these phenomena

Although detailed molecular mechanisms of the embryonic rejuvenation process are still enigmatic, a possible explanation may be the dilution of damaging molecules inside the cells (Figure 4). During cleavage, the total volume of the original embryo is conserved. However, because each cell develops its own maintenance and repair machinery, the ability to clear damage may increase. If we assume that damage from the original parental germ cells is distributed among the newly dividing cells, this could suggest a mechanism where cell‐specific maintenance pathways are able to better clear damage, resulting in a global decrease in deleterious molecules and a subsequent reduction in the biological age readout of the embryo. However, it should be noted that epigenetic clocks do not show a reduction in biological age during cleavage and early stages of embryogenesis. Such reduction is observed only in the later stages, hinting that additional mechanisms, such as the growth of cell mass and/or unequal separation of damage among daughter cells, may define the rejuvenation event observed. Recent development of the first single‐cell epigenetic clock, *scAge*, helped increase the resolution of the above‐mentioned natural rejuvenation process (Trapp et al., 2021), although further studies are needed to both refine the trajectory of epigenetic age changes at the single‐cell level and identify the molecular mechanisms involved.

Identification of this natural rejuvenation event promises several interesting future research directions. There is an obvious need to characterize the multidimensional molecular dynamics at play. Additionally, it will be important to characterize the biological mechanisms related to the subsequent increase in age shortly after the “ground zero” time point, but before birth. This will help to uncover whether there are biological processes in charge of age acceleration of organisms, or if aging is simply a manifestation of the accumulation of damage. If there are specific processes that contribute to age acceleration in the later development of the embryo, it is possible these processes can be inherently perturbed to help slow down aging. Another potential application would be to target the “ground zero” and elucidate the underlying mechanisms behind this natural rejuvenation process, in order to possibly reverse biological age in adult cells. This might be achieved by accentuating the biological rejuvenation phenomenon during early development in order to “begin aging” at a lower biological age (Gladyshev, 2021). Some preliminary studies may support this idea: by growing mice from embryonic stem cells passaged many times in culture, researchers achieved successful elongation of lifespan with low incidence of disease, compared with the same cells that were not successively passaged (Munoz‐Lorente et al., 2019).

### 2.3 Other potential rejuvenation strategies

Although the three categories mentioned above cover the majority of known rejuvenation therapies, this categorization is far from exhaustive, and many other promising strategies may pass the criterion of biomarker‐validated age reversal across several biological modalities. From the strategies analyzed here, damage dilution appears to be a plausible mechanism underlying some rejuvenation interventions. Moreover, it follows that clearance of particularly abundant forms of damage is expected to decrease the age of biological systems in which this damage accumulated.

On the cellular or tissue level, this idea of damage clearance within the cell is closely linked to developing strategies involving senescent cell clearance. It has been recently shown that the removal of senescent cells is associated with improved physiological responses and restoration of organ function, as well as with lifespan extension (Baar et al., 2017; Childs et al., 2017; Pignolo et al., 2020; Xu et al., 2018; Zhu et al., 2015). Of note, a set of mouse models were recently developed where senescent cells expressing p16^Ink4a^ cells can be selectively eliminated (Baker et al., 2016; Demaria et al., 2014). Using this model to clear senescent cells results in reversal of certain aging phenotypes in several tissues, including skeletal muscle and eyes (Baker et al., 2011). Alternative methods include a set of pharmaceutical perturbations called senolytics, which have been reported to ameliorate age‐related functional decline in the nervous and cardiovascular systems, and reduce the age‐related mortality pertinent to coronavirus in mouse models (Camell et al., 2021; Gasek et al., 2021; Xu et al., 2018; Zhu et al., 2015). Interestingly, intermittent administration of these drugs has been shown to reduce side effects to adjacent cells (Kirkland & Tchkonia, 2020). It would be interesting to apply molecular aging biomarkers to these sets of interventions, in order to quantitatively assess their systemic effect. One challenge for this senescence‐clearing strategy is to achieve high selectivity while not disturbing key physiological functions, as there is presently no clear borderline to distinguish senescent from non‐senescent cells along a very dynamic spectrum. Additionally, many senescent cells still are responsible for key biological processes such as wound healing and cellular reprogramming (Demaria et al., 2014; Mosteiro et al., 2016, 2018). It remains unclear how biological age of tissues or adjacent single cells changes as a result of senescence‐clearing therapies.

There are also other strategies, most prominently represented by the SENS (Strategies for Engineered Negligible Senescence) approach, which seeks to remove seven broad categories of damage (de Grey et al., 2002; De Grey & Rae, 2007). In future, it will be interesting to determine whether these approaches work, and how they are related to age reversal as quantified by a diverse set of robust aging biomarkers.

### 2.4 Common features of rejuvenation interventions

**FIGURE 5:**
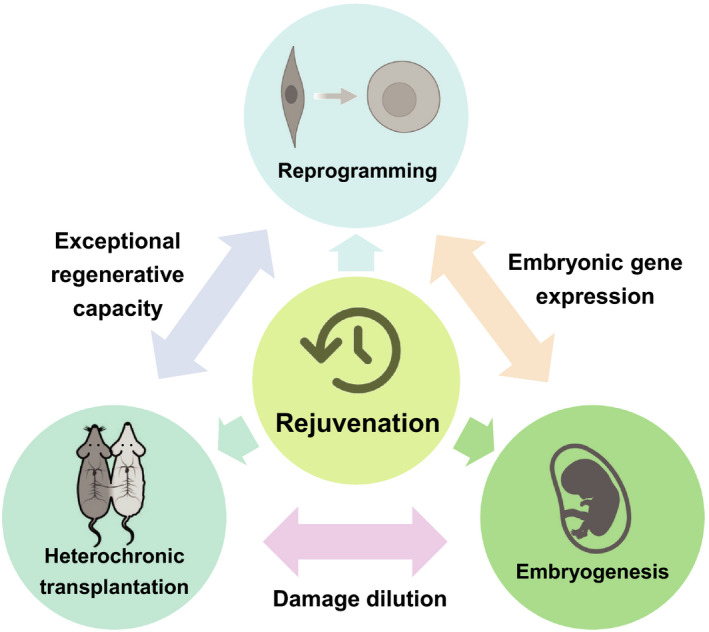
Relationships between different rejuvenation paradigms. Schematic of the connections between three putative rejuvenation strategies. Embryonic rejuvenation and heterochronic transplantation share a common potential mechanism (damage dilution), and embryonic rejuvenation and reprogramming factor expression both involve common changes in gene expression. Additionally, heterochronic transplantation and reprogramming both result in a significant elevation of regenerative capacity. Harnessing the connections between current and future rejuvenation therapies may lead to a more comprehensive framework of rejuvenation, which could enable its eventual systemic application in humans

It is a reasonable assumption that putative “youthful components” are needed if one wants a rejuvenation intervention to be applied to adult individuals. In the case of heterochronic transplantation, these components are the young tissues, cells, and circulating molecules; with reprogramming, they consist of Yamanaka factors identified based on embryo‐specific gene expression; and in the case of the natural “ground zero” process, critical changes in the intra‐ and extra‐cellular environment in embryonic cells during a certain stage of development may be responsible for the observed epigenetic changes and age reversal. In this regard, it would again be useful to explore the orthogonality of reprogramming, in order to deconvolute the effects of de‐differentiation and age reversal (Buganim et al., 2014). Likewise, it would be helpful to determine whether damage dilution‐related effects could be recapitulated by inducing specific gene programs. Indeed, it may also be possible to increase the expression of maintenance pathways in cells, thereby improving damage‐clearing capacity and lowering biological age. This may also reveal if some “youthful” genes are inhibited by the deleterious waste that accumulates in late life, and if we can reverse this process by artificially inducing an environment where damage can be effectively cleared in adult organisms. Commonalities between the rejuvenation therapies discussed in this work are shown in Figure 5.

Rejuvenation strategies have much in common with existing longevity interventions. Although most longevity interventions do not have the ability to systemically reverse biological age like rejuvenation therapies, they serve to attenuate certain age‐related hallmarks, with important effects such as reduced presence of senescent cells and increased stem cell pool size and functionality. This implies that there may be common mechanisms that work to ameliorate specific aging hallmarks, and it would be helpful if common signatures can be developed from omics approaches to identify changes related to age reversal resulting from these therapies. A more important feature common to the existing and potential rejuvenation interventions is the exceptional enhancement of regenerative capacity. Expression of reprogramming factors resulted in the regrowth of neurons, while heterochronic parabiosis was related to the proliferation of liver cells and increased muscle stem cell function. Since embryonic tissues are also highly regenerative, it would be interesting to further investigate the relationship between cell differentiation, regenerative capacity, and biological age.

Overall, rejuvenation interventions open exciting opportunities to reverse biological age of individuals, thereby extending lifespan and health span. However, much of the biological underpinnings of rejuvenation remain enigmatic, and the current side effects induced by some of these interventions (particularly reprogramming) currently prevent their translational application. Current approaches such as reducing the dosage and closely monitoring vital parameters can help reduce these adverse effects. However, it will be critical in future to eliminate these harmful effects via deep multi‐modal analyses of the common features of current and emerging rejuvenation interventions. As more of these interventions are developed and independently validated based on molecular and physiological aging biomarkers, it will eventually be possible to assess and harness the underlying connections between age‐reversing strategies. Ultimately, this research at the interface of aging, molecular profiling, high‐resolution techniques, and physiological assessments may enable safe and effective application of systemic rejuvenation therapies in humans.

## CONFLICTS OF INTERESTS

The authors declare no conflicts of interest.

## AUTHOR CONTRIBUTIONS

B.Z., A.T., and V.N.G. wrote the manuscript with input from C.K.; B.Z. and A.T. designed figures; B.Z. and V.N.G. conceived the thematic, and V.N.G. supervised the work.
